# Are extensions in paid parental leave associated with lower infant and neonatal mortality in Latin American cities? Evidence from 148 cities in Chile, Mexico, and Colombia (2000–2015)

**DOI:** 10.1016/j.socscimed.2025.117971

**Published:** 2025-03-17

**Authors:** Asiya Validova, Jessica Uruchima, Goro Yamada, Alejandra Vives, Alina Schnake-Mahl, Amélia Augusta de Lima Friche, Ariela Braverman, Brisa Sanchez, Hugo-Alejandro Santa-Ramírez, Laura Baldovino Chiquillo, Marcio Alazraqui, Monica Mazariegos, Mónica Serena Perner, Olga Lucia Sarmiento, Tamara Doberti, Tonatiuh Barrientos-Gutierrez, Waleska Teixeira Caiaffa, Ana V. Diez Roux, Ana Ortigoza

**Affiliations:** a Urban Health Collaborative, Dornsife School of Public Health, Drexel University, United States; b Department of Health Policy and Management, Dornsife School of Public Health, Drexel University, United States; c PolicyLab, Children’s Hospital of Philadelphia, United States; d Pontificia Universidad Católica de Chile, Chile; e Observatory for Urban Health in Belo Horizonte, School of Medicine, Federal University of Minas Gerais, Brazil; f Institute for Community Health, Malden, MA, United States; g Facultad de Medicina, Universidad de Antioquia, Medellín, Colombia; h School of Medicine, Universidad de los Andes, Bogotá, Colombia; i Institute of Collective Health, National University of Lanus, Argentina; j Instituto de Nutrición de Centroamérica y Panamá, Guatemala; k Universidad de Chile, Chile; l Instituto Nacional de Salud Publica, Mexico; m Department of Social and Environmental Determinants for Health Equity, Pan American Health Organization, United States

**Keywords:** Infant mortality, Neonatal mortality, Maternity leave, Paternity leave, Urban health, Interrupted time series analysis

## Abstract

We examined changes in infant and neonatal mortality that occurred after extension in the minimum number of days of paid maternity leave and after the implementation of paid paternity leave in 148 cities using longitudinal city-level data (2000–2015) from Chile, Colombia, and Mexico, compiled and harmonized by the Salud Urbana en America Latina (SALURBAL) study. For Chile we also explored variations in these associations according to the mother’s educational attainment as a measure of family socioeconomic standing. We employed interrupted time series analysis in country-specific models, adjusted by time-variant socioeconomic characteristics such as the percent of the population with secondary education and above, and GDP per capita at the city level.

In Chile, we found modestly steeper declines in infant and neonatal mortality rates after paid parental leave reform in 2011 which combined the extensions in paid maternity leave and the introduction of paid paternity leave. We did not find significant associations between extensions of paid maternity and/or introduction of paternity leave and infant and neonatal mortality trends in Colombia and Mexico. The magnitude of the extension in days of paid maternity leave may be relevant to the impacts on infant and neonatal mortality. Results from this study highlight the potential importance of combined paid maternal and paternal leave policies for reducing infant and neonatal mortality while promoting more egalitarian gender roles in successful child upbringing. This is particularly relevant in the context of highly unequal Latin American cities, where women continue to provide the majority of childcare.

## 1. Introduction

Despite the steady decline in infant mortality in Latin America over several decades, reductions in preventable deaths have stalled since 2005 (CEPAL). This stagnation could be explained in part by the fact that once preventable causes of death are tackled, achieving further reductions requires addressing drivers of mortality related to social inequalities in the population (Ortigoza et al., 2021). Socioeconomic participation of women has been considered key to improving infant health (Varkey and Lesnick, 2010). In previous work, it was shown that higher levels of women’s labor force participation and educational attainment were associated with lower infant mortality in Latin American cities (Ortigoza et al., 2021).

Policies and interventions that address gender inequalities and foster women’s empowerment and socioeconomic development can positively impact women’s and children’s health (Borrell et al., 2014; Ortigoza et al., 2021). One such policy is paid leave, in the form of paid maternity or paternity leave (together referred to as paid parental leave) (Ruhm, 2000; Khan, 2020). Paid parental leave has the potential to reduce disparities in health through different pathways. More time off from paid and unpaid work for women during prenatal and postnatal periods has been linked to lower levels of perinatal complications such as low birth weight (LBW) (Guendelman et al., 2009). Greater engagement in caregiving from both parents has been associated with greater child wellness and survival (Tanaka, 2005; Harrington et al., 2014). Greater women’s income levels as a consequence of a reduced share of time spent in domestic work and the ability to remain and progress in the labor market has been linked to lower infant mortality (Heymann et al., 2017). Protected time off during post-natal period also greatly enhances the probability of initiating and continuing breastfeeding, contributes to better child developmental outcomes due to parental bonding in the critical early months, promotes preventive care and better maternal health behaviors which is positively associated with infant health (Yilmaz et al., 2002; Ogbuanu et al., 2011)

Most of the evidence on the benefits of paid parental leave on infant health is based on European policy models, where paid parental leave programs are generous in time off and monetary coverage (Ruhm, 2000; Olivetti and Petrongolo, 2017). In the United States, where paid family leave (PFL) programs are less extended and limited in time, research also showed that in states where PFL has been implemented such as California, these programs improved child health, and reduced infant hospitalizations for avoidable infections and illnesses, and post-neonatal mortality, after adjusting for maternal and neonatal factors (Lichtman-Sadot and Bell, 2017; Pihl and Basso, 2019; Montoya-Williams et al., 2020). Some US studies comparing paid versus non-paid parental leave showed that although both kinds of leave improve infant health, positive impacts of non-paid leave are only present among high-educated women employed in private sector (Rossin, 2011; Bartel et al., 2019), highlighting the relevance of extensive paid parental programs for reducing socioeconomic disparities in infant health. At least two studies based in low- and middle-income countries have found that longer paid maternal leave was associated with lower infant mortality, but these studies did not include any country from Latin America (Nandi et al., 2016; Puliyel et al., 2021).

Although Latin America has an extensive and longstanding regulatory structure for paid maternity leave, for decades the paid parental leave system in the region has not adequately integrated gender equality (IPC-IG and UNICEF, 2020; Blofield and Touchton, 2020). Since 2000, several regional gender commitments contributed to make significant progress on gender equality and women’s autonomy (ECLAC, 2019). Many Latin American countries have implemented extensions in paid maternity leave by increasing the number of days, the amount of monetary benefit that is covered, or by reducing the employment time required to be eligible for this benefit (IBSDRPA, 2016; ECLAC, 2019). Some countries also introduced paid leave entitlements for fathers, albeit less generous than for mothers (summary of the paid parental leave policies in Latin America is presented in Table A1, Appendix A).

The effect of extensions in paid maternity and paternity leave on infant health remains underexplored in the region, particularly in urban areas (Blofield and Touchton, 2020; Galván et al., 2022) where a large proportion (over 80 %) of the population lives and where large social inequalities persist.

Although cities may offer greater opportunities for women to thrive, certain socioeconomic and demographic characteristics may increase socioeconomic disparities among women in urban areas (Duren et al., 2020). In cities with high levels of poverty and high proportions of women as primary income earners, women with less remunerative jobs may not have the resources to pay for domestic help or caregiving activities (Ferrant et al., 2014). In rapidly growing cities with a significant influx of migrants, incoming families may not be able to rely on extended relatives or other social networks to share domestic and child-rearing activities (King-Dejardin, 2019). Care work can prevent women from accessing the labor market; almost three-quarters of non-paid domestic and care work is performed by women and girls in Latin America (Kim et al., 2021). Paid maternity and paternity leave extensions may also have a larger effect on reducing gender and socioeconomic inequities and on infant health disparities in cities with greater socioeconomic inequity because these policies may allow low-income parents with formal work to access benefits that they would otherwise not be able to afford.

In this paper, we capitalized on differences in the extension and incorporation of paid maternity and paternity leave, respectively, in Chile, Colombia, and Mexico (countries that implemented the extensions in paid maternity leave and introduced paid paternity leave policies during 2000–2015, a period for which mortality data is available) and examined the associations between extending the minimum number of paid maternity leave days and introduction of paid paternity leave, and neonatal and infant mortality rates in cities (aim 1). We then explored variations in these associations by women’s socioeconomic standing (aim 2). We hypothesized that greater extensions in paid maternity leave and implementation of paid paternity leave would be associated with stronger declining trends in infant and neonatal mortality, and that the association would be stronger among women of low socioeconomic position women.

## 2. Methods

### 2.1. Data sources

We used data compiled and harmonized by the Salud Urbana en America Latina (SALURBAL) study (Quistberg et al., 2019). For this study we used longitudinal annual data (2000–2015) on infant and neonatal deaths and live births from 148 cities in three countries: Chile (21 cities), Colombia (35 cities) and Mexico (92 cities).

### 2.2. Outcomes

Our two primary outcomes were yearly city-level infant mortality and neonatal mortality rates from 2000 to 2015. Infant and neonatal mortality are defined as the number of deaths during the first year of life and first 28 days of life, respectively, per 1000 live births. Data for deaths and live births were retrieved from vital statistics registries.

### 2.3. Exposures

Our exposure variable was the national implementation of extensions in the minimum number of days of paid maternity leave, and the introduction of paid paternity leave policies. We assessed paid maternity and paternity leave availability by examining updates in country’s labor code for the period 2000–2015 (Appendix A). Chile introduced two extensions in paid maternity leave: in 2003 maternity leave was increased from 12 weeks to 18 weeks (6 weeks before and 12 weeks after childbirth), and in 2011 pregnant mothers were granted 30 weeks of paid maternity leave, starting six weeks before birth and 24 weeks after (IPC-IG and UNICEF, 2020, Blofield and Touchton, 2020). Simultaneously to this last extension, a mandatory five-day paternity leave was implemented. In Colombia, an increase in paid maternity leave from 12 to 14 weeks occurred in 2011 while the incorporation of paid paternity leave happened in 2002 (8 days). Mexico enacted its first paid paternity leave in 2012 (5 days) (IPC-IG and UNICEF, 2020; Hawley and Carnes, 2021) while 90 days of paid maternity leave remained unchanged for the whole study period.

### 2.4. Covariates

We included other factors that might confound the relationship between paternal/maternal leave policies and infant and neonatal mortality in cities: city level of economic development measured by city gross domestic product (GDP) per capita, city educational attainment measured as percent of population with secondary education, and individual-level maternal education (available only for the case of Chile). Annual subnational GDP per capita data were obtained from Gridded global datasets for the Gross Domestic Product and Human Development Index over 1990–2015 (Kummu et al., 2020). Educational attainment data were retrieved from country censuses for the years and countries where census was available. Data for the remaining years in the study period were estimated through linear interpolation and extrapolation. Supplementary Table A2 (Appendix A) shows census year available in each country and the years of estimation. In the stratified analysis by maternal education in Chile, maternal education data was retrieved from live birth records.

### 2.5. Statistical analysis

We conducted descriptive analyses of trends in infant and neonatal mortality rates and paid parental policy changes during the study period.

To investigate the association between maternity leave extension and the introduction of paid parental leave with infant and neonatal mortality, we employed interrupted time series analysis in country-specific models. All models were subsequently adjusted by time-variant socioeconomic characteristics such as the percent of the population with secondary education and above and city-level GDP per capita. Assuming the response to the implementation of extensions of paid maternity leave might not be immediate, we incorporated a 1-year lag into the analyses (i.e. the post intervention period began one year after the policy change).

To estimate relative changes in infant and neonatal mortality pre and post intervention, we used piecewise Poisson regressions (Naumova et al., 2001) with annual infant or neonatal death counts as outcomes, offset by counts of live births. The Models included a random intercept component for the city, and random slope components for time and used robust variance estimation to account for within-city correlation.


$$\text{log}{\left(\text{deaths}\right)}_{it}=\left(\beta_{0}+b_{0i}\right)+\left(\beta_{1}+b_{1i}\right)\left({\text{pre-intervention}\;\text{period}\;\text{linear}\;\text{spline}\;}_{t}\right)\;+\left(\beta_{2}+b_{2i}\right)\left({\text{first-intervention}\;\text{period}\;\text{linear}\;\text{spline}\;}_{t}\right)\;+\left(\beta_{3}+b_{3i}\right)\left({\text{second}\;\text{intervention}\;\text{period}\;\text{linear}\;\text{spline}\;}_{t}\right)\;+\beta_{4}\left({\text{secondary}\;\text{education}\;}_{it}-{\bar{\text{secondary}\;\text{education}\;}}_{i}\right)\;+\beta_{5}\left(\text{log}{\left(GDP\right)}_{it}-{\bar{\text{log}(GDP)}}_{i}\right)$$

In the model above, *i* represents city, and *t* represents time. Linear splines were constructed based on the number and timing of each policy implementation or extension (StataCorp, 2023). For the case of Chile with two policy changes of paid maternity leave, three splines were used to represent the period before the first policy change, the period between the first and second policy changes, and the period after the second policy extension. We refer to these periods as the pre-intervention, first-intervention, and second-intervention periods, respectively. For the rest of the countries with either one policy extension for paid maternity leave or just the implementation of paid paternity leave, we used two splines denoting the pre-intervention and post-intervention periods. Linear splines were constructed such that when used in the regression, their coefficient measures the slope for their respective period (StataCorp, 2023). Each coefficient represents then the annual relative change in mortality for the respective intervention period. Differences between spline coefficients represent the relative difference in changes in mortality between intervention periods. For example, *β*_2_ shows the annual relative change in mortality during the first-intervention period, and *β*_2_ – *β*_1_ shows the relative difference in changes in mortality between the first-intervention and pre-intervention period.

For aim 1, we examined the association between the implementation of extensions in paid maternal leave and infant/neonatal mortality separately in Chile and Colombia. Since in Chile simultaneous changes in both paid maternity and paternity leave occurred in 2011, the effects of maternity leave extension could not be separated from the effects of introduction of paternity leave. Cities in Mexico were excluded from the maternity leave analysis because there was no maternal leave policy change during the study period.

To examine the association between the implementation of paid paternity leave and infant/neonatal mortality, we included cities in Colombia and Mexico - where they were implemented in 2002 and 2012, respectively (see Appendix A, Table A1). (In Chile the implementation of paid paternity leave in 2011 was examined jointly with the extension of maternity leave in the same year as noted above).

For aim 2, we only included cities in Chile, because mortality data disaggregated by maternal educational status was not available for the other countries. We conducted analysis stratified by mother’s level of education (less than secondary education vs secondary and above) and included interactions between the spline components and maternal education in the models (Appendix A, Equation (A3)).

### 2.6. Sensitivity analysis

To check the robustness of the results, for Colombia we restricted the sample to 16 cities for which vital statistics registries presented good quality of death registry based on a previous analysis of adult mortality (Ortigoza et al., 2021). We assumed that cities with good levels of registration for adult deaths (coverage of 90 % or above) may also have better reporting of deaths among infants.

## 3. Results

### 3.1. Descriptive statistics

Table 1 reports the average rates and relative change in infant and neonatal mortality for 2000–2015 in three countries and the summary statistics for the exposure variables and covariates included in the analysis. The average infant mortality rate in Chile (7.8 per 1000 live births) was almost half the rate in Colombia (14.0) and Mexico (13.7); similar differences were observed in average neonatal mortality rates. From 2000 to 2015, cities in Colombia showed the largest reduction in both outcomes (approximately 47 % reduction in infant and neonatal mortality) compared to more moderate decreases in Chile (24.3 % decline in IMR and 9.6 % decline in NMR) and in Mexico (25.6 % and 10.9 % decline, respectively). All countries had 84 days of paid maternity leave in the beginning of the study period (2000). The increase in the minimum number of days of paid maternity leave over the study period was 14 days in Colombia and 126 days in Chile. Introductions of paid paternity leave consisted of 5 days on average (4.67, Table 1). Chile had the largest city GDP per capita and the highest proportion of population with secondary education (Table 1).

### 3.2. Paid maternity and paternity leave in Chile

**Fig. 1.**
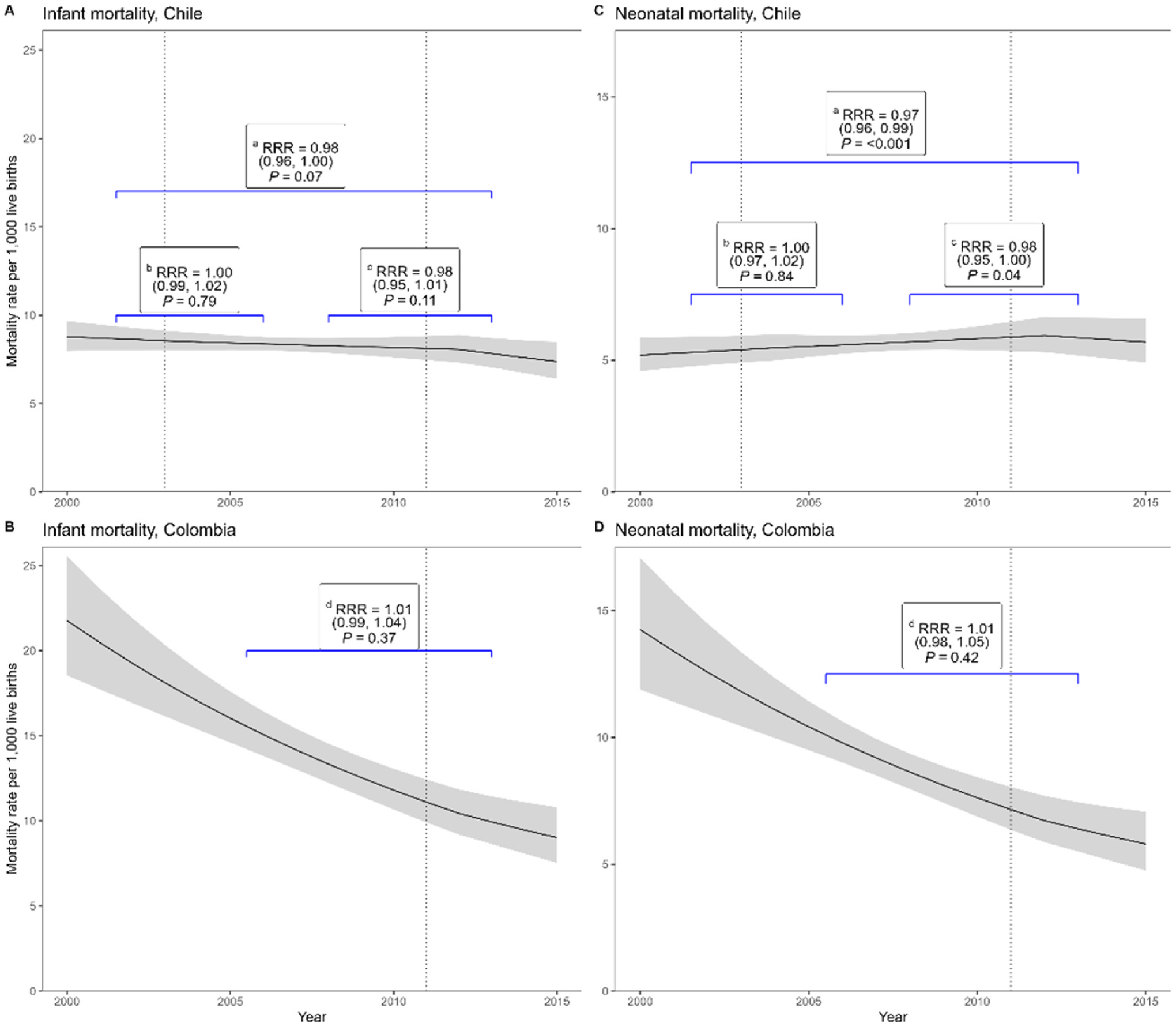
Trends in infant and neonatal mortality rates in cities pre and post interventions and changes associated with extension in paid maternal and paternal leave in Chile and Colombia (2000–2015). **Notes:** Labelled brackets denote the relative difference in mortality trend pre and post intervention, 95 % CI, p-value The second-intervention period for Chile denotes the effect of two policies, an increase in maternal leave and the implementation of paternal leave. For Chile: ^a^Second-intervention vs pre-intervention period. ^b^First-intervention vs pre-intervention period. ^c^Second-intervention vs first-intervention period. For Colombia: ^d^ post vs pre-intervention period.

Results from time series analysis examining the association between extension of paid maternity and paternity leave and infant and neonatal mortality are summarized in Fig. 1 and Table B1 Appendix B. Compared to the baseline period, a marginally significant 2 % acceleration of the already declining trend (a 2 % steeper decline) in infant mortality was observed in Chilean cities one year after the second extension in paid maternity leave (resulting in overall 126 days extension in paid maternity leave) and the introduction of paid paternity leave (5 days) in 2011, after accounting for changes in city socioeconomic characteristics [IMRR: 0.98, 95 %CI: 0.96, 1.00] (Fig. 1 and Table B1, Appendix B). We also observed a 3 % steeper decline in neonatal mortality one year after the extension of paid maternity leave and introduction of paid paternity leave in 2011 compared to the baseline period [NMRR: 0.97, 95 % CI: 0.96, 0.99] (Fig. 1 and Table B1, Appendix B), after adjusting for city socioeconomic characteristics. No effects were observed for the first intervention (increase in maternity leave from 84 to 126 days in 2003).

Results from time series models that examined differential association of paid parental leave with infant and neonatal mortality by levels of maternal education (aim 2) in Chile showed no difference in change in mortality trend across strata of maternal education after extensions in paid maternity leave and introduction of paid paternity leave (relative difference in change, secondary vs. less than secondary 0.98 (CI:0.94, 1.01)) (Fig. 3).

### 3.3. Paid maternity and paternity leave in Colombia

**Fig. 2.**
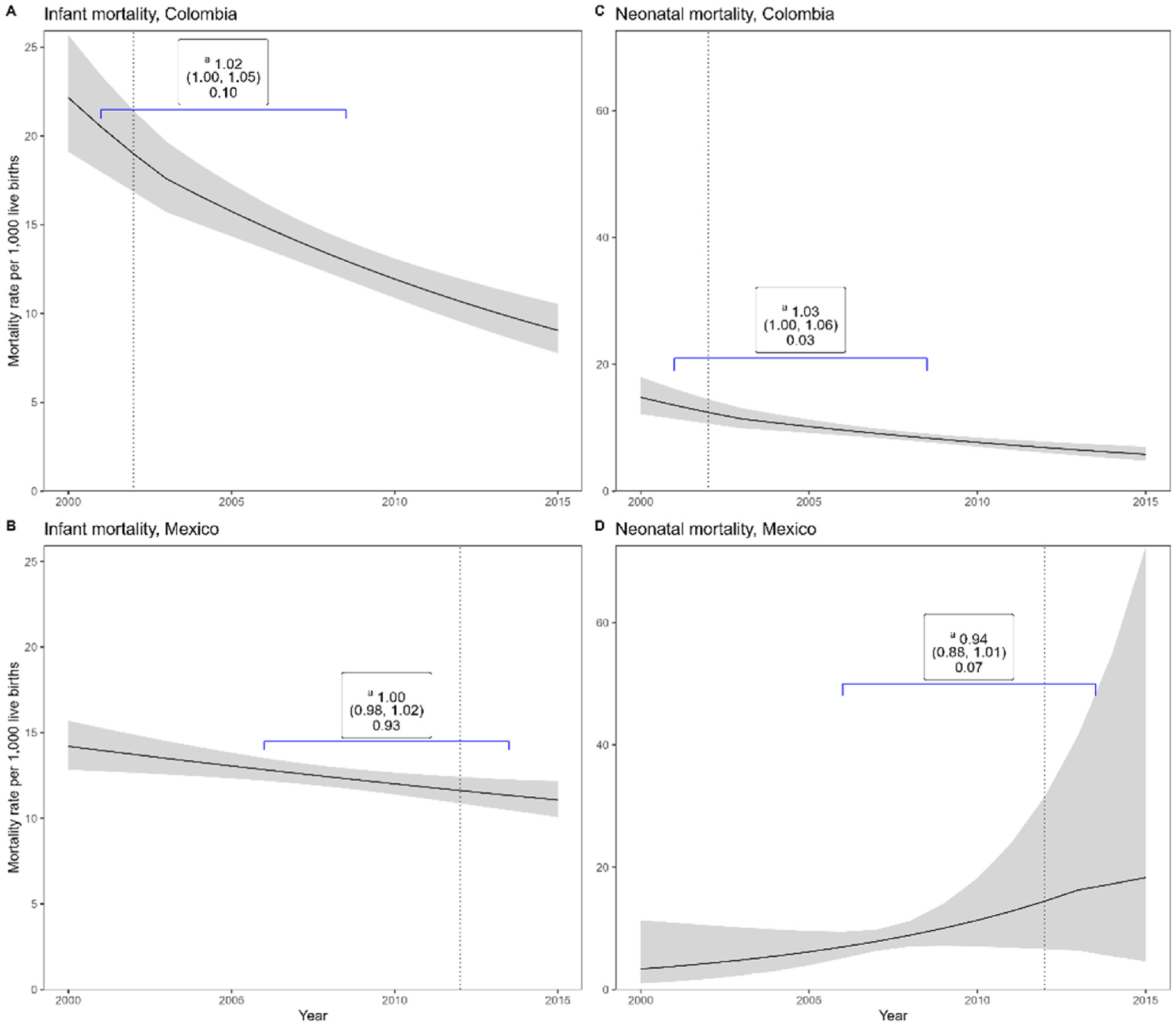
Trends in infant and neonatal mortality rates in cities pre and post interventions and changes associated with extension in paid paternal leave in Colombia and Mexico (2000–2015). Notes:Labelled brackets denote the relative difference in changes in mortality, 95 % CI, p-value. ^a^ Post intervention vs pre-intervention period.

**Fig. 3.**
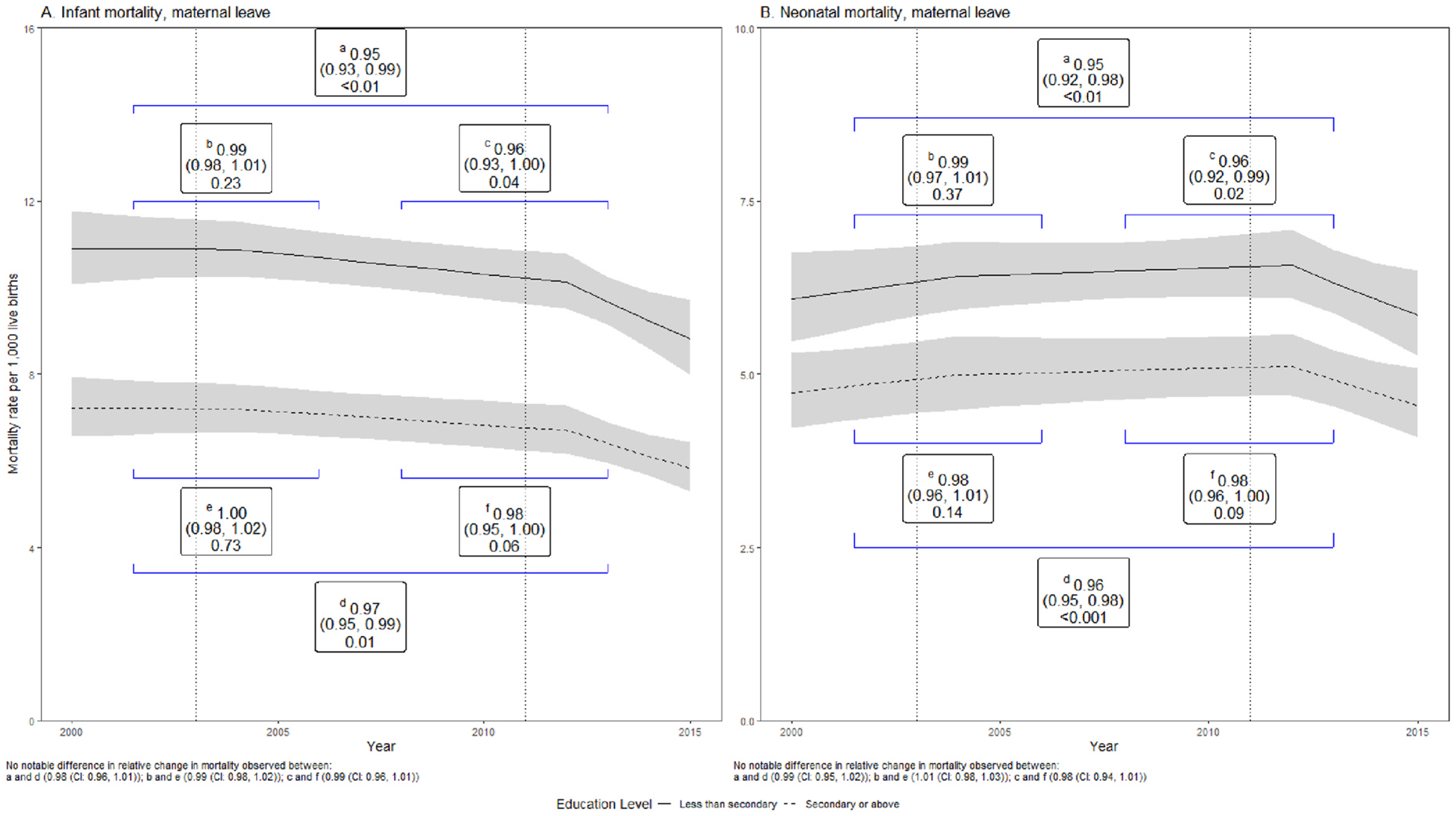
Trends in infant and neonatal mortality rates in cities pre and post interventions and changes associated with extension in paid maternal and paternal Leave by mother’s educational level in Chile 2000–2015. **Notes:** Labelled brackets denote the relative difference in change in mortality, 95 % CI, p-value. The second-intervention period denotes the effect of two policies, an increase in maternal leave and the implementation of paternal leave. ^a^Less than secondary, second-intervention vs first-intervention period. ^b^Less than secondary, first-intervention vs pre-intervention period. ^c^Less than secondary, second-intervention vs first-intervention period. ^d^Secondary or above, second-intervention vs first-intervention period. ^e^Secondary or above, first-intervention vs pre-intervention period. ^f^Secondary or above, second-intervention vs first-intervention period.

In cities from Colombia, infant and neonatal mortality did not show statistically significant changes in trends after the extension in the minimum days of paid maternity leave from 84 to 98 days in 2011 in the fully adjusted model (Fig. 1 and Table B1, Appendix B). There were also no significant changes in infant mortality rates [IMRR: 1.02, 95 %CI: 1.00, 1.05] or neonatal mortality rates [NMRR: 1.03, 95 %CI:1.00,1.06] after one year of the introduction of paid paternity leave (8 days) in 2002 (Fig. 2 and Table B2, Appendix B). We found similar results in sensitivity analysis on the restricted sample of 16 cities (results not shown).

### 3.4. Paid paternity leave in Mexico

In cities from Mexico there was a 6 % steeper decline in neonatal mortality one year after the introduction of paid paternal leave (NMRR: 0.94, 95 %CI: 0.88, 1.01] but the change was not statistically significant. There was no change in infant mortality after the introduction of paid paternity leave [IMRR: 1.00, 95 %CI: 0.98, 1.02] (Fig. 2 and Table B2, Appendix B).

## 4. Discussion

In this study we examined changes in infant and neonatal mortality that occurred after extensions in days of paid maternity leave and after the implementation paid paternity leave in 148 cities from Chile, Colombia, and Mexico. In Chile, we found modestly steeper declines in infant and neonatal mortality rates after paid parental leave reform in 2011 which combined the extensions in paid maternity leave and the introduction of paid paternity leave. We did not find associations between extensions of paid maternity leave and introduction of paid paternity leave and infant and neonatal mortality trends in Colombia, where both interventions occurred separately in time (2011 and 2002, respectively). We also did not find statistically significant changes in infant and neonatal mortality after the implementation of paid paternity leave in Mexico, where days of paid maternity leave remained unchanged.

Our study showed that concurrently increasing the duration of paid maternity leave and implementation paid paternity leave policies, as in the case of Chile, may have the greatest potential to reduce infant and neonatal mortality in cities. Maternity leave secures women’s income during leave and allows women to remain in the labor force after childbearing contributing to narrowing the wage gender gap and maintaining women’s socioeconomic status, which in turn can improve overall household wealth and consequently child well-being (Chatterji and Markowitz, 2012). Additionally, paid maternity leave secures time during the pregnancy and postnatal period to recover from delivery, which promotes longer breastfeeding period and access to health check-ups and immunization visits which translates into better birth and child health outcomes (Navarro-Rosenblatt and Garmendia, 2018; Montoya-Williams et al., 2020; Galván et al., 2022). By facilitating greater participation of fathers in childcare and of mothers in the labor market paid paternity leave may leverage the effect of paid maternity leave (Borrell et al., 2014; Patnaik, 2019). Since we did not find an association between implementation of paid paternity leave and infant or neonatal morality in Mexico and Colombia where extensions in paid maternity were unchanged, further research is needed to better understand how the length of paid paternity leave may impact neonatal or infant mortality separately or in interaction with paid maternity leave.

The magnitude of the extension in days of paid maternity leave could be also relevant to the impacts on infant and neonatal mortality. In Chile we observed declines in infant and neonatal mortality associated to the interventions only after the generous extension in days of paid maternity leave that occurred in 2011 (which involved an overall 126 days extension since the baseline period) while a 42 days extension in 2003 did not result in significant changes in mortality outcomes. Similarly, the smaller increase in paid maternity leave in Colombia (14 days) showed no impact. Further analyses are needed to determine whether there is a threshold in the minimum number of days of paid maternity leave above which significant health impacts may be seen.

The heterogeneity of findings across countries also calls for better understanding of the specific characteristics of the policies adopted by each country. Maternity leave policies may vary in terms of the length of pre- and post-natal periods (not examined separately in this study) which can potentially affect infant health through separate mechanisms. More generous coverage of pre-natal days can be linked to better infant health through better follow-up and timely treatment of pregnancy conditions, adequate birthweight and overall mother’s physical and mental health during pregnancy and delivery (Heymann et al., 2017, IPC-IG and UNICEF, 2020). Longer post-natal coverage may not only benefit mother’s post-delivery health but also contribute to longer breastfeeding, better child healthcare and increases infant immunization uptake (Ray et al., 2010; Navarro-Rosenblatt and Garmendia, 2018, IPC-IG and UNICEF, 2020). Further research including both pre- and post-natal period would allow a better understanding of specific mechanisms contributing to better child health and well-being.

In this study we mainly examined how changes in the length of paid maternity leave and the introduction of paid paternity leave (characteristics related to the ‘intensity’ of the policies studied) could allow improvements in infant and neonatal health outcomes. Additionally, many of the extensions in paid maternity leave and introduction of paid paternity leave policies in the countries under study were done along with changes in the necessary requisites for receiving these benefits, which could explain further impacts in health outcomes through an expansion in the policy coverage (Appendix A). The case of Chile is notable, because during the last paid maternity leave extension in 2011 the additional 84 days granted after 126 days of exclusive paid maternity leave were given with a flexible schedule (named as ‘postnatal parental rest’), so that mothers could make use of them in the totality or share its last 35 days with the father if he was also employed. Although there is no publicly available data on the extension in policy coverage and uptake of this ‘postnatal parental rest’ extension, it could have contributed to the beneficial effects observed in neonatal and infant outcomes after the parental policy reform in 2011 in Chile.

Our study had several limitations. First, since paid parental policies are benefits granted to formal workers, it is possible that associations shown in this study may be confounded by the unmeasured level of informal employment in cities, leading to an underestimation of the true impact of the policies explored. Due to lack of subnational and longitudinal estimation of informal employment in cities, we were not able to account for the level of formal or informal employment in our models. Consideration of level of informal work in cities may be particularly relevant for Colombia and Mexico where labor informality rates are recognized to be high, although difficult to estimate and assess over the period under study. Recent statistics showed that workers in informal employment represented 28 % of Chile’s total employment in 2019 (WIEGO Statistical Brief, 2022), while in Mexico labor informality was estimated at 56.2 % of the total employed population for 2019, and in Colombia labor informality between November 2023 and January 2024 was estimated at 55.7 % (Espejo, 2022, Departamento Administrativo Nacional de Estadística - Gran Encuesta Integrada de Hogares, 2024).

Second, the interrupted time series approach implemented in this study may not take into consideration the impact of simultaneous adoption of other policies or local interventions directly or indirectly affecting infant health, through better health care access for infants or state-funded childcare. Over the last decade, many governments in the region implemented social protection policies which aimed to increase investment in health and increase insurance coverage that contributed to reduction in maternal and child mortality rates in countries that enacted these policies (GTR, 2017; García and Vaeza, 2023). Except in the case of Chile where we studied both paid paternity leave and maternity leave changes, we have not examined other concurrent policies that could benefit child health. Furthermore, we did not investigate the magnitude of changes in the leave-taking after the extensions in the days of maternity leave and introduction of paternity leave in Latin American region, where the low uptake by fathers of shared parental leave is a common challenge (Fernandez, 2022).

Third, the interrupted time series analysis only allowed us to assess immediate changes in health outcomes, so we were not able to measure the long-term impact of these policies on child health. Further longitudinal studies are needed to examine if policies had a sustained long-term impact on infant health.

To our knowledge this is the first study to examine infant health impacts of extensions in paid maternity leave and implementation of paid paternity leave on critical outcomes such as infant and neonatal mortality in a large number of Latin American cities. Results from this study highlight the potential importance of combined paid maternal and paternal leave policies for reducing infant and neonatal mortality while promoting more egalitarian gender roles in successful child upbringing. This is not only relevant in the context of highly unequal Latin American cities, where addressing gender bias in childcare is still a pending issue, but also to the United States, where limited paid leave policies and large income and gender inequalities in health in many US cities bear important similarities to the Latin American ones. Moreover, as the impacts of leave policies are culturally mediated (Mitchell, 2004) and Hispanic workers in US have shown lower rates of paid leave access than White non-Hispanic counterparts (Stearns, 2015, Jou et al., 2018; Bartel et al., 2019) further evidence on processes that support greater policy coverage and uptake in Latin America could serve as valuable lessons for advancing the parental leave policy landscape and reducing health inequalities in the United States.

## Figures and Tables

**Table 1:** Paid parental policies, socioeconomic characteristics of cities and city mortality rates by countries during the study period (2000–2015).

Variable	Chile (21 cities)	Colombia (35 cities)	Mexico (92 cities)
**Outcomes**			
2000 infant mortality rate	9.00	19.40	16.14
2015 infant mortality rate	6.82	10.09	12.00
Average Infant mortality rate per 1000 live births^a^, (SD)	7.83 (0.61)	13.97 (2.90)	13.67 (1.30)
Relative change in IMR (2000–2015), %	− 24.25	− 47.98	− 25.63
2000 neonatal mortality rate	5.60	12.67	8.59
2015 neonatal mortality rate	5.06	6.63	7.66
Average Neonatal mortality rate,^a^ (SD)	5.33 (0.22)	8.98 (1.81)	8.93 (1.42)
Relative change in neonatal mortality (2000–2015), %	− 9.63	− 47.71	−10.87
**Exposures**			
Duration of paid maternity leave in 2000 (in days)	84	84	84
Increase in days of maternity leave (2000–2015)	126	14	0
Duration of paid paternity leave in 2000 (in days)	0	4	0
Increase in days of paternity leave (2000–2015)	5	4	5
**Covariates**			
Average Yearly GDP per capita, USD^a^	19,681.85 (14,834.24)	9365.50 (3683.58)	15,119.94 (12,632.28)
Average Population with secondary education and above^a^(%)	49.29 (7.84)	41.72 (7.84)	30.17 (7.92)

Notes:

Sample includes 2359 data points from 148 cities.

Mortality rates are calculated using annual vital registry data from country ministry of health or national statistics office.

Exposure information gathered from IPC-IG and UNICEF, 2020, Blofield and Touchton (2020).

Annual subnational GDP per capita data were obtained from Gridded global datasets for the Gross Domestic Product and Human Development Index over 1990–2015 (Kummu et al., 2020).

Secondary education obtained from country censuses. Non-census years are estimated using linear interpolation and extrapolation.

a Average measures for cities included in analyses over 2000–2015.

## Data Availability

Data will be made available on request.

## Data statement

The SALURBAL project welcomes queries from anyone interested in learning more about its dataset and potential access to data. To learn more about SALURBAL’s dataset, visit https://drexel.edu/lac/or contact the project at salurbal@drexel.edu.

## Appendix A

**Table A1:** Paid parental programs in Latin American countries included in the SALURBAL study^#^

Country	Year of update in paid maternity leave (days approved)^a^	Year of change in paid paternity leave (days approved)^a^
Argentina	1978 (90)	1974 (2)
Brazil	1988 (120)	1988 (5)
Chile	1972 (84); 2003 (126); 2011 (210)	2011 (5*) **
Colombia	1990 (84); 2011 (98); 2017 (126)	2002 (8*) **
Costa Rica	1996 (120)	none
Guatemala	1993 (84)	2001 (2) **
Mexico	1996 (84); 2019 (98)	2012 (5*) **
Panama	1995 (98)	2017 (3) **
Peru	1996 (90); 2015 (98)	2009 (4) **; 2018 (10)
El Salvador	1972 (84); 2015 (112)	2013 (3) **

Notes:

# Parental programs are those officially recognized by the national law/regulation in each country.

* Corresponds to working days and not continuous days.

** There was no paid paternity leave before that time.

a Years of leave update present since year 2000. Excludes extension of days for special cases like premature newborns, newborns with diseases that require special needs, or multiple gestations.

### Summary of progress in paid maternity and paternity leave in Latin America

Over the last two decades almost all Latin American countries enacted policies that increased the duration of paid maternity leave which now ranges from the lowest 12 weeks in Ecuador, Guatemala, and Nicaragua to the highest of 30 weeks in Chile (Galván et al., 2022). The presence and changes in maternity and paternity leave policies are heterogeneous across the region. Argentina and Brazil for example, have had similar legislation regarding paid maternity and paternity leave for more than 30 years, while other countries like Colombia or Chile have experienced more than one extension in the days of paid maternity leave since 2000. Most of these countries have implemented paid paternity leave since 2000 (Chile, Colombia, El Salvador, Guatemala, Mexico, Panama, and Peru) although the size of the benefit has been small compared to maternity leave.

Below we provide an overview of the paid parental leave policies under examination for Chile, Colombia, and Mexico – countries that underwent policy changes during the period of the study 2000–2015.

### Chile

In 1972 Labor code established 84 days of paid maternity leave (42 days prenatal or 42 postnatal), with no paid paternity leave. In 2003 (Decrero con Fuerza de Ley 1/2003), the New Labor Law extended paid maternity leave to 126 days (42 pre and 84 postnatal) and established 5 working days (at least 7 continuous days) of paid post-natal leave to fathers working in governmental/public dependencies (Art 195).

An executive order in November 2011 extended this paid paternity leave to all workers. In that same year, the Law 20545/2011 Art 197 incorporated 12 more weeks (84 days) of paid leave during the postnatal period under the clause of ‘postnatal parental rest’ (‘descanso parental posnatal’). This leave is conceded to mother, adding a total of 210 days of paid maternity leave (42 prenatal and 168 days postnatal). Paid days of leave under the ‘postnatal parental rest’ period can be used by the mother as a whole or she can choose to share 35 of the 84 days conceded (the last 5 weeks of the 12 weeks) with the father if he is also employed. These 5 weeks can be conceded completely to fathers or distributed among both parents (let say 2.5 each, or 2 mother 3 father).

### Colombia

Since 1990 the labor code established 84 days of paid leave to mothers (14 days pre and 70 postnatal) and only 4 days of paternity leave in the case the father (but not the mother) was employed and contributing to the Social Security (Sistema General de Seguridad Social en Salud) (Ley 50/1990). In the case both parents were employed and contributing to the Social Security, fathers could have 8 days of paternity leave. In any case, for father acceding to this paid leave, they should have been contributing to the Social Security System for more than 100 weeks before the newborn’s delivery.

In 2002 Law 755/2002 (Ley Maria) conceded 8 working days of paid leave to all fathers no matter whether the mother was also employed and contributing to the Social Security or how much time the father has been contributing to the Social Security System.

In 2011, Law 1468/2011 extended 12–14 weeks of paid maternity leave (a total of 98 days distributed in 14 days pre and 84 postnatal). In 2017, Law 1822/2017 extended maternity leave from 14 to 18 weeks (126 days total, 14 pre and 112 postnatal).

### Mexico

Previous to 1996, maternity leave was of a total of 8 weeks (56 days total, 28 pre and postnatal, respectively). In 1996, a reform of The Federal Law of Labor (art 170-II) extended paid maternity leave to a total of 12 weeks (84 days total, 42 days pre and postnatal, respectively). In this reform no paid leave for fathers was included.

In 2012 through a modification of Art 170 (DOF 30–11-2012), allowed to roll over 4 of the 6 weeks of the prenatal period to the postnatal time. It also extended same rights of accessing to paid leave to mothers adopting children. In this reform of the Law that Art 132–25 was added, through which paid paternal leave was established on 5 days. Since then, no further extension on paid paternity leave has occurred.

In 2019, an extension from 12 to 14 weeks of paid maternity leave was approved (98 total days, 49 per and postnatal, respectively). In this stage, mothers were also able to roll over 6 out of the 7 weeks of the prenatal period (up to 42 days) to the postnatal time.

**Table A2:** Censuses used for the linear interpolation and extrapolation of educational attainment

Country	Census Years
Chile	2002, 2017
Colombia	2005, 2018
Mexico	2000, 2010

Eq. (A3).

in the model (Appendix 1, Table A3)

$$\text{log}{\left(\text{deaths}\right)}_{\text{it}\;}=\left(\beta_{0}+b_{0i}\right)+\left(\beta_{1}+b_{1i}\right){\left(\text{pre}-\text{intervention}\;\text{period}\;\text{linear}\;\text{spline}\right)}_{t}+\left(\beta_{2}+b_{2i}\right){\left(\text{first}-\text{intervention}\;\text{period}\;\text{linear}\;\text{spline}\right)}_{t}\;+\left(\beta_{3}+b_{3i}\right){\left(\text{second}-\text{intervention}\;\text{period}\;\text{linear}\;\text{spline}\right)}_{t}+\beta_{4}{\left(\text{maternal}\;\text{education}\right)}_{t}\;+\beta_{5}(\text{pre}-\text{intervention}\;\text{period}\;\text{linear}\;\text{spline}\;\times \;\text{maternal}\;\text{education})\;+\beta_{6}(\text{first}-\text{intervention}\;\text{period}\;\text{linear}\;\text{spline}\;\times \;\text{maternal}\;\text{education}\;)+\beta_{7}(\text{second}-\text{intervention}\;\text{period}\;\text{linear}\;\text{spline}\;\times \;\text{maternal}\;\text{education})\;+\beta_{8}\left(\text{log}{\left(GDP\right)}_{it}\bar{\text{log}{\left(GDP\right)}_{i}}\right)$$

## Appendix B

**Table B1:** Relative change in infant and neonatal mortality associated with extension in paid maternal leave with 1-year lag (country-specific models, 2000–2015)

	Chile (n = 336 from 21 cities)		Colombia (n = 560 from 35 cities)	
	Unadjusted	Adjusted	Unadjusted	Adjusted
Annual relative change in infant mortality^1^				
Pre-intervention period	0.99 (0.97,1.00) 0.01	0.99 (0.98,1.01) 0.28	0.95 (0.95,0.96) <0.001	0.94 (0.92,0.96) <0.001
First-intervention period^2^	0.99 (0.98,0.99) 0.00	0.99 (0.98,1.01) 0.41	0.97 (0.94,0.99) 0.01	0.95 (0.92,0.98) <0.01
Second-intervention period^3^	0.97 (0.94,0.99) 0.01	0.97 (0.94,1.00) 0.05	–	–
Relative difference in changes in infant mortality^4^				
First-intervention vs pre-intervention period	1.00 (0.99, 1.02) 0.78	1.00 (0.99, 1.02) 0.79	1.01 (0.99, 1.04) 0.33	1.01 (0.99, 1.04) 0.37
Second-intervention vs pre-intervention period	0.98 (0.96, 1.00) 0.07	0.98 (0.96, 1.00) 0.07	–	–
Second-intervention vs first-intervention period	0.98 (0.95, 1.01) 0.11	0.98 (0.95, 1.01) 0.11	–	–
Annual relative change in neonatal mortality				
Pre-intervention period	1.00 (0.99,1.02) 0.74	1.01 (0.99,1.03) 0.20	0.96 (0.95,0.96) <0.001	0.94 (0.92,0.96) <0.001
First-intervention period	1.00 (0.99,1.01) 0.96	1.01 (0.99,1.03) 0.34	0.97 (0.94,1.00) 0.04	0.95 (0.92,0.99) 0.01
Second-intervention period	0.98 (0.96,0.99) <0.01	0.99 (0.97,1.00) 0.12	–	–
Relative difference in changes in neonatal mortality				
First-intervention vs pre-intervention period	1.00 (0.97, 1.02) 0.86	1.00 (0.97, 1.02) 0.84	1.02 (0.98, 1.05) 0.36	1.01 (0.98, 1.05) 0.42
Second-intervention vs pre-intervention period	0.97 (0.96, 0.99) <0.001	0.97 (0.96, 0.99) <0.001	–	–
Second-intervention vs first-intervention period	0.98 (0.95, 1.00) 0.04	0.98 (0.95, 1.00) 0.04	–	–

Notes:

1 Exponentiated coefficients RR/CI/p-values.

2 First intervention in Chile denotes the extension in the number of days of maternity leave in 2003.

3 Second intervention in Chile denotes the combined effect of extension in the number of days of maternity leave and introduction of paternity leave in 2011.

4 Relative difference in mortality trend pre and post intervention, 95 % CI, p-value.

**Table B2:** Relative annual change in infant and neonatal mortality associated with extension in paid paternal leave with 1-year lag (country-specific models, 2000–2015)

	Colombia (n = 560 data points from 35 cities)		Mexico (n = 1463 data points from 92 cities)	
	Unadjusted	Adjusted	Unadjusted	Adjusted
Annual relative change in infant mortality^1^				
Pre-intervention period	0.94 (0.92,0.96) <0.001	0.93 (0.90,0.95) <0.001	0.99 (0.98,1.00) <0.001	0.98 (0.97,0.99) <0.01
First-intervention period	0.96 (0.95,0.96) <0.001	0.95 (0.93,0.96) <0.001	0.99 (0.98,1.01) 0.47	0.98 (0.97,1.00) 0.10
Relative difference in changes in infant mortality^2^				
First-intervention vs pre-intervention period	1.02 (1.00, 1.05) 0.07	1.02 (1.00, 1.05) 0.10	1.00 (0.99, 1.02) 0.67	1.00 (0.98, 1.02) 0.93
Annual relative change in neonatal mortality				
Pre-intervention period	0.92 (0.89,0.95) <0.001	0.92 (0.89,0.95) <0.001	1.13 (0.96,1.33) 0.14	1.13 (0.96,1.33) 0.14
First-intervention period	0.95 (0.92,0.97) <0.001	0.95 (0.92,0.97) <0.001	1.06 (0.85,1.33) 0.62	1.06 (0.85,1.33) 0.62
Relative difference in changes in neonatal mortality				
First-intervention vs pre-intervention period	1.03 (1.00, 1.06) 0.03	1.03 (1.00, 1.06) 0.03	0.94 (0.88, 1.01) 0.07	0.94 (0.88, 1.01) 0.07

Notes:

1 Exponentiated coefficients RR/CI/p-values.

2 Relative difference in mortality trend pre and post intervention, 95 % CI, p-value.
